# Learning in Probabilistic Boolean Networks via Structural Policy Gradients

**DOI:** 10.3390/e27111150

**Published:** 2025-11-13

**Authors:** Pedro Juan Rivera Torres

**Affiliations:** 1 Departamento de Computación y Automatización, Universidad de Salamanca, CB3 0BN Salamanca, Spain; pedro.rivera@usal.es; 2 St. Edmund’s College, University of Cambridge, Cambridge CB2 1TS, UK

**Keywords:** Probabilistic Boolean Network, statistical learning, Learning Probabilistic Boolean Networks

## Abstract

We revisit Probabilistic Boolean Networks as trainable function approximators. The key obstacle, non-differentiable structural choices (which predictors to read and which Boolean operators to apply), is addressed by casting the PBN’s structure as a stochastic policy whose parameters are optimized with score-function (REINFORCE) gradients. Continuous output heads (logistic/linear/softmax or policy logits) are trained with ordinary gradients. We call the resulting model a Learning PBN. We formalize the Learning Probabilistic Boolean Network, derive unbiased structural gradients with variance reduction, and prove a universal approximation property over discretized inputs. Empirically, Learning Probabilistic Boolean Networks approach ANN performance across classification (accuracy ↑), regression (RMSE ↓), representation quality via clustering (ARI ↑), and reinforcement learning (return ↑) while yielding interpretable, rule-like internal units. We analyze the effect of binning resolution, operator sets, and unit counts, and show how the learned logic stabilizes as training progresses. Our results indicate that PBNs can serve as general-purpose learners, competitive with ANNs in tabular/noisy regimes, without sacrificing interpretability.

## 1. Introduction

Conventional Boolean networks are celebrated for interpretability and finite-state dynamics (attractors), but they are usually fixed rule systems. In contrast, artificial neural networks (ANNs) learn end-to-end via gradient descent. This work asks a simple question with practical ramifications:

*Can a Probabilistic Boolean Network be trained to learn like an ANN while retaining logic-level interpretability?*

Two challenges prevent a straightforward “yes.” First, structural choices in a PBN, like selecting which predictors to read and which logical operator to apply, are discrete and non-differentiable. Second, continuous targets (regression, policy values) require mapping Boolean computations to the reals.

Enter our approach: we introduce the Learning PBN (LPBN): each Boolean unit stochastically selects two input bits and a logical operator (AND/OR/XOR/NAND) from categorical distributions. The distributions’ logits are trainable parameters. The Boolean outputs feed simple differentiable heads (linear/softmax/policy). We optimize the heads with ordinary gradients and the structure with REINFORCE using an exponential-moving-average baseline to reduce variance.

This work addresses a key limitation of traditional PBNs: the difficulty in optimizing their non-differentiable structure in data-driven settings. Our objective is twofold: (1) to introduce a differentiable framework that enables learning over PBN structures using policy-gradient methods, thereby improving their trainability, and (2) to extend the utility of PBNs beyond biological modeling into general-purpose interpretable learning systems. The proposed Learning-based PBN (LPBN) combines the logical interpretability of Boolean networks with the optimization capabilities of deep learning, offering a principled approach to structure learning in stochastic logic-based models.

This paper presents the following contributions:

1. A precise mathematical formulation for Learning Probabilistic Boolean Networks.
2. Unbiased structural gradients with practical variance reduction.
3. A universal approximation result over discretized inputs and discussion of approximation bias/variance.
4. A multi-paradigm evaluation vs. ANNs across classification, regression, clustering-via-representation, and RL.
5. Ablation studies (bins, operators, width) and interpretability analyses via extracted rules.

## 2. Related Work

### 2.1. Boolean and Probabilistic Boolean Networks

Kauffman’s Boolean networks (BNs) [1] established discrete, rule-based models of gene regulation whose long-run dynamics concentrate on attractor sets (limit cycles and fixed points) that have been linked to cellular “phenotypes”. Probabilistic Boolean Networks (PBNs) generalize BNs by assigning a distribution over predictor functions per node; the induced stochastic switching makes the network a finite-state Markov chain whose stationary measure allocates probability mass to attractor basins [2,3,4]. Analytical treatments connect network structure to steady-state behavior, enabling computation of attractor probabilities and residence times [5]. This paper builds on that line by treating the selector distributions in a PBN as learnable objects, optimized from task loss—classification error, regression risk, or return—rather than being fixed from prior biological knowledge.

### 2.2. Intervention and Control in PBNs

Because PBNs define a Markov chain, optimal interventions [4,6,7] can be framed as Markov decision/control [8] problems over the state space or reduced abstractions. Early work examined structural interventions—altering predictor sets or their selection probabilities—to shift stationary behavior [4]. Subsequent methods considered context-sensitive PBNs and derived intervention policies that minimize the long-run probability of undesirable states [6] as well as state space reduction to make intervention tractable [7]. Optimal-control formulations using dynamic programming further grounded the design of intervention policies [8]. Compared to these control-theoretic approaches, our learning-centric treatment uses policy-gradient style updates to fit selector distributions directly to supervised and reinforcement objectives, while preserving the interpretability of discrete predictors and attractor structure.

### 2.3. Learning with Discrete Choices: Gradient Estimators and Relaxations

Training models with discrete latent decisions is classically approached with score-function estimators (REINFORCE; [9]), often stabilized by baselines/advantages. Modern variance-reduction techniques, REBAR [10] and RELAX [11], and continuous relaxations, Gumbel-Softmax/Concrete [12,13], enable lower-variance or differentiable surrogates for categorical sampling. Straight-through estimators provide a pragmatic alternative when exact gradients are unavailable [14]. Our LPBN training objective instantiates this toolbox directly: selector logits for (i) which input each node reads and (ii) which Boolean operator it applies are updated with advantage-weighted score-function gradients; optional temperature-controlled relaxations can replace hard sampling for end-to-end differentiability.

### 2.4. Neuro-Symbolic and Logical Learners

Several propositional/relational learners connect logic with gradient-based training. The Tsetlin Machine learns propositional clauses with bandit dynamics and has shown competitive accuracy on tabular/text tasks while remaining interpretable [15]. Neural Logic Machines [16] and Differentiable Proving [17] explore differentiable reasoning over rules, whereas Logic Tensor Networks [18,19] integrate logical constraints within neural architectures. LPBNs are complementary: they maintain strictly Boolean internal computations (AND/OR/XOR/NAND over binarized features) but learn the composition by optimizing selector distributions. This keeps interpretability at the node/logic level while enabling multi-task training akin to neural networks.

### 2.5. Reinforcement Learning and Deep RL Foundations

Policy-gradient RL provides the theoretical underpinning for optimizing stochastic decision rules with respect to expected return [20]. Benchmark environments such as CartPole from OpenAI Gym [21] standardize evaluation of controller learning. In our DRL experiments, LPBNs act as stochastic policies over discrete logical features of a discretized observation, trained with advantage-weighted score-function updates; neural baselines use shallow or two-layer MLP policies trained with the same on-policy objective, isolating the effect of the representation (logical vs. continuous).

### 2.6. Clustering and Unsupervised Evaluation

For unsupervised structure learning, we follow established external and internal indices. The Adjusted Rand Index (ARI) measures agreement between predicted partitions and ground truth, corrected for chance [22]. The Silhouette coefficient [23] and Calinski–Harabasz index [24] quantify cohesion-vs.-separation without labels. When we compare LPBN-derived features to MLP embeddings under k-means, ARI provides the primary, label-aware criterion, with Silhouette/CH as label-free diagnostics.

### 2.7. Final Remarks

Beyond classical interventions and attractor control, recent research has addressed several advanced aspects of PBN control. For example, minimum observability problems aim to identify the smallest subset of nodes whose states must be monitored to ensure system controllability [25]. Robust set stability refers to conditions under which a set of network states remains stable under probabilistic perturbations and intervention noise [26]. Related to this is the notion of set stabilization, which focuses on driving the system toward a desirable subset of states with bounded error under stochastic dynamics [27]. These developments are especially relevant in practical applications such as gene therapy or therapeutic reprogramming, where interventions must remain effective under uncertainty. Our work, while not focused on the control problem directly, complements this literature by proposing a data-driven way to learn the probabilistic structure of Boolean dynamics, which may serve as a foundation for future interpretable control policies.

## 3. Materials and Methods

Given $x\;\epsilon \;R^{d}$, standardize each of the dimensions and partition *B* bins. Concatenate one-hot bin indicators to obtain

(1) $$z\;\epsilon {\left\{0,1\right\}}^{m},\;m=dB,$$

a Boolean substrate on which the network will operate. Equation (1) models the output of a Boolean node *x_i_* in a probabilistic Boolean network. In traditional PBNs, each node is governed by a set of possible Boolean functions, and one is selected probabilistically at each update. Here, instead of fixed candidate functions, the stochasticity is embedded in the logic structure itself, defined by a distribution over logical triplets (*a*,*b*,*o*). The expectation in Equation (1) thus captures the average output of node *x_i_* over the stochastic structure space, akin to the ensemble behavior of classical PBNs with randomly selected update functions. Equation (1) encodes the system as a one-hot binary vector input to the PBN. This serves as the input substrate analogous to the Boolean state vector used in standard PBN formalism. Discretization trades smoothness for interpretability and lets us compose exact logical operations.

### 3.1. Stochastic Logical Units

The LPBN has *N* Boolean units. Unit *n* selects two input positions and one operator:

(2) $$A_{n}\sim \pi_{n}^{A},\;B_{n}\sim \pi_{n}^{B},\;O_{n}\sim \pi_{n}^{O},$$

with categorical distributions

$$\pi_{n}^{A}(i)=\frac{e^{\alpha_{n,i}}}{\sum_{u=1}^{m}e^{\alpha_{n,u}}},\;\pi_{n}^{B}(j)=\frac{e^{\beta_{n,j}}}{\sum_{v=1}^{m}e^{\beta_{n,v}}}$$

given the sampled ($a_{n},b_{n},o_{n},$), the unit emits

$$h_{n}=g_{O_{n}}\left(z_{a_{n}},z_{b_{n}}\right)\in \left\{0,1\right\},$$

where

$$a_{n},b_{n},o_{n},\sim \pi_{n}$$

where $a_{n},b_{n},o_{n}$ are input indexes and $o_{n}\;$ is a logic operator (AND, OR, XOR, etc.). Each node *n* in the network samples its two input connections independently; $A_{n}$ and ${\;B}_{n}$ are node indices drawn from categorical distributions ${\;\pi }_{n}^{A}$ and ${\;\pi }_{n}^{B}$, respectively. These distributions assign probabilities over the set of possible source nodes in the previous layer. The operator $O_{n}$ is sampled from $\pi_{n}^{O}$, a categorical distribution over a fixed set of Boolean Operators O={AND, OR, XOR, NAND,…}. $\pi_{n}$ is the combined policy for structural selection for unit *n*, α, β, o are categorical distributions on inputs and outputs, $z_{n}=z_{a}op\;z_{b}$ is the result of applying operator $o_{n}$ over two selected bits, and g∈{AND, OR, XOR, NAND}. Collect *h* = ${\left(h_{1},\ldots ,h_{N}\right)}^{T}\in {\left\{0,1\right\}}^{N}$. Given the sampled inputs $A_{n}$ and ${\;B}_{n}$ and operator $O_{n}$, the output of node *n* is computed as $x_{n}=O_{n}(z_{A_{n}},z_{B_{n}})$, where $z_{A_{n}},z_{B_{n}}$ are the activations of the selected input nodes.

Each unit learns what to look at (two discretized features) and how to combine them (logic). As training proceeds, the categorical posteriors sharpen, revealing a rule-like structure.

#### Output Heads

- Binary classification: $\hat{y}=\;\sigma (w^{T}h+b)$, cross-entropy loss.
- Multiclass: $\hat{y}=softmax(Wh+b)$, negative log-likelihood.
- Regression: = logits $\phi_{a}=\sum_{n}\theta_{n},\;ahn,\;\pi (\alpha |z)=softmax(\phi ),\;objective\;J\;=\;E[R]$.

where $\theta_{n}$ denotes the set of parameters that govern the structure distributions for node *n*, including the logits for ${\;\pi }_{n}^{A},$ ${\;\pi }_{n}^{B},$ ${\;\pi }_{n}^{O}$.

### 3.2. Learning Rules

Continuous parameters (*w*, *b*, *W*, θ) use standard gradients of the task objectives (e.g., SGD or Adam). Structural logits (α, β, γ) are trained with a score-function estimator. Let ***F*** denote the scalar objective (use ***F*** = −L for supervised tasks; **F** = **R** for RL) and *b* a baseline. Let L denote the number of input features and *R* the number of output bits for the Boolean Network. Each output unit computes a binary value based on its logic structure and input activations. The vector of output values produced by the Boolean Network is denoted by ***R*** $\in {\left\{0,1\right\}}^{R}$. Then

(3) $$\nabla_{\alpha_{n,i}}E[F]=E[(F-b)\nabla_{\alpha_{n,i}}log{\;\pi }_{n}^{A}(A_{n})]=E[(F-b)(1\{A_{n}=i\}\;-\;\pi_{n}^{A}(i))].$$

Similar expressions follow and hold for β,γ.  These updates are applied per mini-batch (supervised) or per trajectory (RL).

### 3.3. Variance Reduction and Stabilization

We model a Learning Probabilistic Boolean Network as a stochastic computation graph over discrete selectors. For an input of $x\in {\left\{0,1\right\}}^{d}$, every node *n* $\in \left\{1,\ldots ,\;N\right\}$ draws a selector triple

(4) $$S_{n}=(A_{n},B_{n},O_{n})\;\sim \;\pi_{\theta_{n}}(\cdot |\;x)$$

where $A_{n},B_{n},\;\in \;\{1,\ldots ,\;d\}$ are index input literals and $O_{n}\in \{AND,\;OR,\;XOR,\;NAND\}$ is a Boolean operator. With the (deterministic) Boolean map $h_{n}={op}_{O_{n}}(x_{A_{n}},\;x_{B_{n}})$ and a linear readout $f_{w}(h)\;=\;w^{T}h+b$, we can define a task loss of $l(f_{w}(h),y)$. The training objective is the selector-marginalized risk

(5) $$J(\theta ,w)=E_{(x,y)\sim D}E_{S\sim \pi_{0(\cdot |x)}}[l(f_{w}(h(x,S)),y))].$$

### 3.4. Unbiased Gradient for Discrete Selectors (REINFORCE)

Differentiating *J* with respect to selector parameters θ yields the score-function estimator from Williams (1992) [9]:

(6) $$\nabla_{\theta }J=E_{D,S}[l(f_{w}(h),y)].$$

Replacing the intractable expectation by Monte Carlo samples ($x_{i},y_{i},S_{i}$) gives an unbiased gradient, but with high variance.

### 3.5. Control-Variate Baseline and “Advantage”

Let *F* = $l(f_{w}(h),y)$ and *g* = (*F* − *b*)$\nabla_{\theta }\;log\;\pi_{\theta }(S|x)$ with any baseline *b* independent of the sampled *S*. Because E[$\nabla_{\theta }\;log\;\pi_{\theta }(S|x)$] = 0, we have that $E[g]\;=\nabla_{\theta }J\;$ (unbiased). This is the standard REINFORCE-with-baseline form used in both the supervised and RL training. For a shared scalar baseline, the variance-minimizing constant is

(7) $$b^{*}=\frac{E[F||\nabla_{\theta }\;log\;\pi_{\theta }(S|x){\left|\right|}_{2}^{2}]}{E[||\nabla_{\theta }\;log\;\pi_{\theta }(S|x){\left|\right|}_{2}^{2}]}$$

and it is obtained by differentiating Var[*g*] with respect to *b*. In practice, we approximate it online by an exponential-moving average (EMA), b←βb+(1−β)F with β∈[0.9, 0.99], which substantially reduces gradient variance without bias.

### 3.6. Entropy Regularization to Prevent Premature Collapse

LPBN selectors are categorical; early collapse $\pi_{0}(\cdot |x)\to one-hot$ kills exploration and yields vanishing gradients on unchosen options. Following maximum-entropy RL, we add an entropy bonus

$$J_{\lambda }(\theta ,w)=J(\theta ,w)+\lambda E_{x}[H(\pi_{0}(\cdot |x))],H(\pi )=-\sum_{s}\pi (s)\;log\;\pi (s),\;$$

such that

(8) $$\nabla_{\theta }J_{\lambda }=E[(F-b)\nabla_{\theta }\;log\;\pi_{\theta }(S|x)]+\lambda E[\nabla_{\theta }H(\pi_{\theta })]$$

Annealing λ↓0 over training preserves exploration initially and allows confident selection later.

### 3.7. Rao-Blackwellization over Independent Factors

When selectors factorize across nodes, $\pi_{\theta }(S|x)=\prod_{n}\pi_{\theta_{n}}(S_{n}|x)$, replacing *F* with the conditional expectation, $\tilde{F_{n}}=E[F\;|\;x,\;S_{n}]$ in the update for $\theta_{n}$ yields a Rao-Blackwellized estimator with provably lower variance

(9) $$E[(F-b)\nabla_{\theta_{n}}\;log\;\pi_{\theta_{n}}(S_{n}|x)]=E[(\tilde{F_{n}}-b)\nabla_{\theta_{n}}\;log\;\pi_{\theta_{n}}(S_{n}|x)]$$

and Var[R-B] ≤ *Var*[*original*] by the law of total variance. In practice, we approximate $\tilde{F_{n}}\;$ with a small learned critic conditioned on node-local context (a one-layer regressor), which is the same control-variate idea as below.

### 3.8. Continuous Relaxations and Low-Variance Control Variates (REBAR/RELAX)

For categorical selectors, the Gumbel-Max trick and the Concrete/Gumbel-Softmax relaxation can be exploited to form a differentiable surrogate $\tilde{S_{\tau }}$ with a temperature of τ>0. REBAR constructs an unbiased gradient through the combination of a reparametrized path through the relaxation with a control-variate correction

(10) $$\nabla_{\theta }J\;\approx \underset{low-variance\;pathwise}{\underbrace{\nabla_{\theta }E[\tilde{F_{\tau }}]}}+\underset{score-function\;correction}{\underbrace{E[(F-\tilde{F_{\tau }}{)\nabla }_{\theta }\;log\;\pi_{\theta }(S|x)]}}$$

where $\tilde{F_{\tau }}$ is the loss evaluated on the relaxed sample with common random numbers (same Gumbel noise) to maximize the correlation with F. RELAX generalizes this by learning a control variate $c_{\phi }(\tilde{S_{\tau }},x)$ to minimize the variance of the resulting estimator; the final gradient remains unbiased while often achieving order-of-magnitude variance reductions on discrete graphs.

### 3.9. Straight-Through Estimators

The straight-through estimator (ST) enables gradient-based optimization over non-differentiable binary or discrete functions by approximating gradients during backpropagation. While the forward pass uses hard thresholding (e.g., sign or step functions), the backward pass substitutes their gradients with continuous surrogates—commonly the identity or clipped derivatives of a smooth approximation. This heuristic stems from early work by Hinton and was formalized by [14] as a low-variance, computationally efficient proxy. The core justification is that, under certain conditions, the direction of the ST update correlates positively with the true expected gradient, enabling convergence in stochastic optimization despite non-differentiability.

A well-known limitation of the ST is its biased gradient estimation, which arises from ignoring higher-order interactions between discrete variables and the loss surface. This bias can accumulate when binary units saturate (i.e., become nearly deterministic), undermining learning. Entropy regularization directly addresses this by encouraging intermediate activation probabilities, thus keeping the network in a regime where the surrogate gradients remain informative. This approach has proven effective in learning binary representations in variational autoencoders [12] and binarized neural networks [28], where entropic penalties or temperature annealing maintain trainability through the ST.

In comparison to alternative estimators—such as REINFORCE or score-function gradients—the ST provides substantially lower variance and faster convergence. REINFORCE, though unbiased, is highly noisy and often infeasible for high-dimensional or deep architectures. Other reparameterization tricks for discrete variables, like Gumbel-Softmax or Concrete distributions, offer more principled solutions, but often suffer from temperature tuning issues, mismatch at inference time, or incompatibility with hard logic operators. In logic-based networks like LPBNs, the deterministic nature of logical gates aligns better with the ST’s forward discreteness and backward differentiability than with continuous relaxations.

Nonetheless, the ST is not universally robust. Its performance degrades when networks collapse to extreme binary probabilities early in training, or when gradient signal vanishes due to poor surrogate choice. These issues can be alleviated through techniques such as entropic smoothing, stochastic dropout, or delayed hardening of outputs via temperature scheduling. Given these mitigations, and the increasing evidence of STE’s success in hybrid neuro-symbolic systems [29], its adoption in PBN training offers a justified balance of tractability and functional fidelity. This makes it a pragmatic and theoretically sound choice for backpropagation in probabilistic logic networks.

### 3.10. Putting Everything Together for a PBN

For supervised learning, use *F* = −$l(f_{w}(h),y)]$. Update *w* by standard gradients on l. Update selector logits θ with $(F-b)\nabla_{\theta }\;log\;\pi_{\theta }$ (the EMA baseline), plus entropy. For a difficult task, replace *F* with REBAR/RELAX control-variate forms. For Reinforcement Learning, let *F* be the (discounted) return or a GAE-style advantage; identical selector updates apply. This matches the policy-gradient framework of [20], with $\pi_{\theta }\;$ the policy over discrete logical features extracted by the LPBN. Under standard Robbins–Monro conditions on step sizes and bounded second moments of *g*, the stochastic approximation on *θ* converges to a stationary point of *Jλ*. In practice, (i) EMA baselines and Rao–Blackwellization/critics reduce gradient variance; (ii) entropy annealing prevents early mode collapse; and (iii) REBAR/RELAX provide the largest additional gains when *F* is sparse or highly non-smooth, exactly the regimes where discrete logical choices are appealing.

### 3.11. Computational Complexity

A forward pass is *O(N)* Boolean ops + *O(N)* dot-product for the head. Sampling from *m*-way and 4-way categoricals per unit is *O(m)* if naive; with alias tables or top-*k* constraints, it is near *O(1)* amortized.

## 4. Model and Objective

Let $x\in {\left\{0,1\right\}}^{d}$ denote a binarized input obtained from raw $R^{d}$ by one-hot quantization or thresholding. An LPBN with *N* internal nodes consists of selector triples

$$S_{n}=(A_{n},B_{n},O_{n}),A_{n},B_{n}\in \{1,\ldots ,d\},O_{n}\in O:\;=\{AND,\;OR,\;XOR,\;NAND\},$$

drawn from a categorical policy $\pi_{\theta_{n}}(\cdot |x)$ with parameters $\theta_{n}$. Given $S_{n},$ node *n* outputs a Boolean feature,

$$h_{n}={op}_{O_{n}}\left(x_{A_{n}},y_{B_{n}}\right)\in \left\{0,1\right\},$$

and we collect $h={\left(h_{1},\ldots h_{N}\right)}^{T}\in {\left\{0,1\right\}}^{N}$. A linear readout produces a task score of $f_{w}(h)\;=\;w^{T}h+b$. For supervised learning with example (*x*, *y*)~*D* and a loss of $l(f_{w}(h),y),$ our risk is

(11) $$J(\theta ,w)=E_{(x,y)\sim D}E_{S\sim \pi_{\theta (\cdot |x)}}[l(f_{w}(h(x,S)),y)].$$

where $D={\left\{(x^{\left(i\right)},y^{\left(i\right)})\right\}}_{i=1}^{N}$ is the dataset of input vectors $x^{\left(i\right)}\in R^{n}$ and the corresponding targets $y^{\left(i\right)}\in Y$, used to compute the expected loss.

For reinforcement learning, $\pi_{\theta }$ is the policy overs selectors, or selector-parametrized action logits, and J(θ) is minus the expected/discounted return. Drawing *S* at each time step yields a PBN, i.e., a finite-state Markov Chain on ${\left\{0,1\right\}}^{d}$ whose kernel is determined by the selector distributions [2,3]. If the selectors are redrawn i.i.d. each step, the chain is homogeneous. A fixed selector draw recovers a deterministic BN. Steady-state and attractor behavior follow the standard Markov Chain theory [4,5,6,7,8].

### 4.1. Expressivity

We will first record the representational capacity of the logical core.

**Lemma 1.** 
*(Functional Completeness): Because {NAND} is functionally complete, any Boolean function g:* $\;{\left\{0,1\right\}}^{m}\to \{0,1\}\;$ *can be realized by a finite acyclic network of 2-input NAND gates. Since*  O  *contains NAND, an LPBN of sufficient width/depth can realize any g on finitely many Boolean literals (including one-hot bins of real features).*

**Proof.** 
A standard result from switching logic: a multi-input AND/OR can be implemented by a binary tree of NANDs; negation is x↦NAND(x,x). For any finite set $X\subset {\left\{0,1\right\}}^{d}$ and any mapping $f^{*}:X\to R$, there exists an LPBN and a readout (θ,w) that matches $f^{*}$ on *X*. We can use Lemma 1 to implement indicator functions for each x∈X via conjunctions of literals, the readout forms $f^{*}(x)=\sum_{x^{\prime }\in \;X}f^{*}(x^{\prime })\;1\{x=x^{\prime }\}.$ □

**Proposition 1.** 
*Let be compact and*$\;f^{*}:K\to R\;$*continuous. For any* ε>0, *there exists a quantizer q:*$\;K\to {\left\{0,1\right\}}^{d}$, *an LPBN over q(x), and a readout* (θ,w,b) *such that*$\;\left|f(x)-f^{*}(x)\right|\leq \varepsilon ,\;\forall \;x\in K.\;$*Proof: For each*$\;x^{\left(i\right)}\in X$, *define K*$\;\subset R^{d}\;$*the indicator:*

(12) $$1_{x^{\left(i\right)}}(x)={⋀}_{j=1}^{d}\tilde{l_{j}}\left(x_{j}\right),\tilde{l_{j}}\left(x_{j}\right)=\left\{\begin{array}{c} x_{j}\;if{\;x}_{j}^{\left(i\right)}=1\\ {\neg x}_{j}\;if{\;x}_{j}^{\left(i\right)}=0 \end{array}\right.$$

i.e., it is the conjunction of literals that match the bits of $x^{\left(i\right)}$. Through Lemma 1, $1_{x^{\left(i\right)}}$ has a realization by NAND gates; chaining conjunctions produces a binary tree whose leaves are literals. Let the LPBN nodes compute $1_{x^{\left(i\right)}}(x)$ for all values of i. Choose the readout weights $w_{i}=f^{*}(x^{\left(i\right)})$ and b = 0. Then $f_{w}(h(x))=\sum_{i}f^{*}(x^{\left(i\right)})1_{x^{\left(i\right)}}(x)=f^{*}(x)$ and b = 0. Then $f_{w}(h(x))=\sum_{i}f^{*}(x^{\left(i\right)})1_{x^{\left(i\right)}}(x)=f^{*}(x)$ on X.

**Corollary 1.** 
*(Uniform approximation on compact subsets of* $\;R^{d}$ *): Let K* $\subset R^{d}\;$ *be compact and* $\;f^{*}\;:K\to R\;$ *is continuous. For any*  ε>0,  *there is a finite partition {* $C_{1},\ldots ,C_{M}$ *} of K into axis-aligned rectangles, a binarization of q:* $\;K\to {\left\{0,1\right\}}^{d}\;$ *that encodes the cell index in one-hot form, an LPBN over q(x), and a readout such that* $\;\left|f_{w}(h(q(x)))-f^{*}(x)\right|\leq \varepsilon \;\forall x\in K.\;$ *Proof: Continuity on compact K implies uniform continuity. Hence there is a grid partition such that the oscillation from* $\;f^{*}\;$ *on each cell C_m_ is at most*  ε.  *Define q to output the one-hot code of the active cell. For each m, the indicator* $\;1_{C_{m}}(q(x))\;$ *is a conjunction of the one-hot- bits that correspond to C_m_ and is therefore realizable by NAND gates (see Lemma 1). Set the readout to* $\;w_{m}=arg\;{mean}_{x\in C_{m}}f^{*}(x)$ *, that is any representative value in the cell. Then* $\;\left|f_{w}(h(q(x)))-f^{*}(x)\right|\leq \varepsilon \;$ *on every cell, establishing uniform approximation. Each LPBN node computes a named Boolean operator on explicit literals; proofs above constructively identify which literals (or cell bits) are used. This yields circuit-level explanations unavailable in standard continuous neurons.*

### 4.2. Unbiased Gradients for Discrete Selectors

Define the instantaneous feedback as $F:\;=l(f_{w}(h),\;y)$ (for supervised) or F: = −G, where *G* is the return (for RL). By the log-derivative trick (REINFORCE), the gradient with respect to selector parameters is

(13) $$\nabla_{\theta }J=E_{D,S}[F\;\nabla_{\theta }\;log\;\pi_{\theta }(S|x)]$$

A Monte Carlo simulation estimate of the samples $\left(x_{i},y_{i},S_{i}\right)$ is therefore unbiased. The readout parameter *w* admits ordinary gradients $\nabla_{w}J=E_{D,S}[\nabla_{w}l]$ because $f_{w}$ is differentiable.

**Lemma 2.** 
*(baseline and advantage): Let b be any random variable independent of the sampled S conditional on x (e.g., a moving average). Then*

(14) $$\nabla_{\theta }J=E_{D,S}[(F-b)\;\nabla_{\theta }\;log\;\pi_{\theta }(S|x)]$$

meaning that subtracting *b* preserves unbiasedness while reducing variance [9,20]. The variance-minimizing constant baseline is

(15) $$b^{*}=\frac{E[F||\nabla_{\theta }\;log\;\pi_{\theta }(S|x){\left|\right|}_{2}^{2}]}{E[||\nabla_{\theta }\;log\;\pi_{\theta }(S|x){\left|\right|}_{2}^{2}]}$$

**Proof.** 
Score has a zero mean. Note that $E[Z\;|\;x]=\sum_{s}\pi_{\theta }(s\;|x)\nabla_{\theta }\;log\;\pi_{\theta }(s\;|x)=\sum_{s}\nabla_{\theta }\pi_{\theta }(s\;|x)=\nabla_{\theta }\sum_{s}\pi_{\theta }(s\;|x)=\nabla_{\theta }1=0.$ Therefore, E[Z]=0.

1. Unbiasedness: Since *b* is independent of *S* given *x*, $E[g_{b}]=E[(F-b)\;Z]=E[FZ]-E[bZ]=E[FZ]-E[bE[FZ]-E[FZ]-E\{bE[Z\;|\;x]\}=E[FZ]$, so subtracting *b* does not change the expectation.
2. Optimal constant: Since the mean $E[g_{b}]\;$ is independent of *b*, minimizing Var($g_{b})$ is equivalent to minimizing $E{\left||g_{b}|\right|}^{2}:E{\left||g_{b}|\right|}^{2}=E[{\left(F-b\right)}^{2}\;{\left||Z|\right|}^{2}]$. Consider the function $\phi (b)=\;E[{\left(F-b\right)}^{2}\;{\left||Z|\right|}^{2}]$. It is a strictly convex quadratic in *b* (unless ${\left||Z|\right|}^{2}$= 0 a.s.). Differentiating and setting the derivative to zero, $\phi^{\prime }=\;{-2(E[F-b)}^{2}\;{\left||Z|\right|}^{2}]=0\;⟹{E[F||Z||}^{2}]=b\;E[{\left||Z|\right|}^{2}]$. If $E{\left||Z|\right|}^{2}>0$, the unique minimizer is $b^{*}=\frac{E[F{\left||Z|\right|}^{2}]}{E[{\left||Z|\right|}^{2}]}$, which minimizes $E{\left||g_{b}|\right|}^{2}$ and also Var($g_{b})$.□

### 4.3. Entropy Regularization

Adding $\lambda E_{x}[H(\pi_{0}(\cdot |x))]$ with $H(\pi )\;=-\;\sum_{s}\pi (s)\;log\;\pi (s)\;$ yields $\nabla_{\theta }(J\;+\;\lambda E_{x}[H])=E[(F-b)\nabla_{\theta }\;log\;\pi_{\theta }]+\lambda E[\nabla_{\theta }H(\pi_{\theta })]$ which prevents premature selector collapse and is standard in policy-gradient practice.

For categorical selectors, Concrete/Gumbel-Softmax relaxations [10,12] enable a low-variance pathwise term coupled with a score-function correction to keep the estimator unbiased (REBAR/RELAX). Denoting the relaxed sample by $\tilde{S_{\tau }}$ (temperature τ),

(16) $$\nabla_{\theta }J\;\approx \underset{reparametrized}{\underbrace{\nabla_{\theta }E[\tilde{F_{\tau }}]}}+\underset{score-function\;correction}{\underbrace{E[(F-\tilde{F_{\tau }})\nabla_{\theta }\;log\;\pi_{\theta }(S\;|x)]}}$$

where you can optionally replace $\tilde{F_{\tau }}$ by a learned control variate, $c_{\phi }(\tilde{S_{\tau }},x)$ (RELAX) to further reduce variance without bias.

### 4.4. Variance Reduction via Rao-Blackwellization

If selector factors $\pi_{\theta }(S\;|x)=\prod_{n=1}^{N}\pi_{\theta_{n}}(S_{n}\;|x)$ across nodes, then

(17) $$\nabla_{\theta_{n}}J=E[\underset{=:\tilde{F_{\tau }}}{\underbrace{E[F\;|\;x,\;S_{n}]}}\nabla_{\theta_{n}}log\;\pi_{\theta_{n}}(S_{n},x)]\;$$

where replacing F by $\tilde{F_{\tau }}$ in node-local updates is a Rao-Blackwellization that strictly reduces variance. In practice $\tilde{F_{\tau }}$ can be approximated by a small critic (regressor) on node-local context-equivalently, a control-variate architecture [12,13].

### 4.5. Convergence: Stochastic Approximation

Let $g_{t}$ represent any of the unbiased (or asymptotically unbiased) gradient estimators above, with $E[g_{t}\;|\;F_{t}]=\nabla J(\theta_{t})$ and $E\left[{\left\|g_{t}\right\|}^{2}\;|\;F_{t}\;\right]\leq C(1+{\left\|\nabla J(\theta_{t})\right\|}^{2})$. With step sizes $\eta_{t}>0$ satisfying $\sum_{t}\eta_{t}=\infty$, $\sum_{t}{\eta_{t}}^{2}<\infty$ (Robbins-Monro conditions), the iterates $\theta_{t+1}=\theta_{t}-\eta_{t}g_{t}$ converge to the set of stationary points of *J* almost surely under standard regularity (e.g., boundedness via projection) [30,31]. Entropy annealing preserves these conditions. For RL, the same conditions apply to the negative-return objective with policy-gradient estimators [9,20].

### 4.6. Reinforcement Learning: Policy-Gradient Form for LPBNs

Consider an episodic MDP with trajectories $\tau =(s_{0},a_{0},\ldots ,s_{T})$ and return $G(\tau )=\sum_{t=0}^{T-1}\gamma^{t}r_{t}.$ Let $\pi_{0}$ be the LPBN policy (e.g., selectors parametrize action logits). The policy-gradient theorem specialized to LPBN yields

(18) $$\nabla_{\theta }J(\theta )=E_{\tau \sim \pi_{\theta }}\left[\sum_{t=0}^{T-1}A_{t}\nabla_{\theta }\;log\;\pi_{\theta }(a_{t}\;|\;s_{t})\right]$$

where $A_{t}\;$ is any advantage signal (for example, $G_{t}-b(s_{t})$); the proof follows the REINFORCE identity plus the Markov property [9,20]. Thus, the same estimators and variance-reduction devices from Section 4.3 and Section 4.4 apply verbatim to LPBN policies in DRL.

### 4.7. Attractors and Stationary Behavior Under Learning

For a fixed selector policy $\pi_{\theta }$, the induced PBN on the discrete state space X has a transition matrix $P_{\theta }$. When $P_{\theta }$ is irreducible and aperiodic, it admits a unique stationary distribution $\mu_{\theta }\;$(Perron-Frobenius theorem); $\mu_{\theta }$ concentrates mass on attractor basins in proportion to expected residence times. Learning alters $\theta ⟼P_{\theta }⟼\mu_{\theta }$. Under ergodicity and mild smoothness of $\pi_{\theta ,\;}\;$ perturbations $\left\|\theta -\theta^{\prime }\right\|$ yield Lipschitz-continuous changes in $P_{\theta }$ and consequently in $\mu_{\theta }$ (Markov Chain perturbation), which justifies gradient-based structural interventions [4,6,7,8] and our selector-probability updates: by increasing expected task reward (or decreasing loss), they reweight the attractor landscape toward a desirable steady-state behavior.

### 4.8. Practical Implications of Sample-Efficiency and Identifiability

Because node outputs are binary, gradients pass only through selector probabilities, not through real-valued non-linearities. This produces low-dimensional stochastic updates with strong regularization by discreteness, often improving small-data generalization. Entropy bonuses and control variates are crucial to avoid premature determination of selectors, which could stall exploration and harm identifiability of useful logic features [9,10,11,12,13,20].

## 5. Experimental Setup

### 5.1. Tasks and Data

We evaluate LPBNs against standard ANN baselines on six canonical tasks, spanning supervised, unsupervised, and control settings.

1. Binary Classification (synthetic): Inputs $x\in R^{8}$ are drawn i.i.d. from a standard normal; labels are generated by a linear score $s=w^{T}x+\epsilon ,$ with $\epsilon \;\sim \;N(0,\;\sigma^{2})$ and y=1{s≥0}. Train/validation/test split is 70/10/20.
2. Regression (synthetic): Same covariates as (1), with targets $t=w^{T}x+\epsilon ,$ with $\epsilon \;\sim \;N(0,\;\sigma^{2}$). Split as above.
3. Clustering (gaussian blobs): We sample K = 3 isotropic Gaussian clusters in $R^{4}$ with angularly separated means (pairwise angle 120°), then hide the labels after training to compute external clustering indices on embeddings.
4. Reinforcement Learning (LineWorld): A 1D chain of length L = 9 with terminal states at the ends; reward +1 on the right terminal, −1 on the left, 0 otherwise. Episodes terminate upon reaching a terminal; horizon 40.
5. Deep RL (CartPole): Classical inverted pendulum control with continuous state ($x,\;\dot{x},\;\theta ,\;\dot{\theta }$) and binary actions (left/right). Episodes terminate on pole angle or cart position violation; horizon 280.
6. Text (negation sensitivity): Synthetic sentiment sentences of the form “the product is [not] [intensifier] word.” Words are drawn from composition induced by negation.

All synthetic generators (1–3, 6) are fixed across seeds by publishing the PRNG seeds used in the experiments. For control tasks (4–5), we use deterministic physics and environment seeds, reported with results.

### 5.2. Preprocessing and Binarization

Before binning, continuous features are standardized to zero mean and unit variance on the training split only; the same affine map is applied to validation and test. We then perform equal-width binning per feature into *B* bins (default *B* = 5; ablation in Section 6.4 uses *B* ∈ {3, 7}), and one-hot encode each bin, yielding a binary vector $q(x)\;=\;{\left\{0,1\right\}}^{D}$ with D =B·d. For CartPole we discretize each state component within a fixed physical range into *B* = 8 bins per variable. LineWorld states are already one-hot.

### 5.3. Models

#### 5.3.1. LPBN

An LPBN with *N* nodes samples selector triples $S_{n}=(A_{n},B_{n},O_{n})$ with $A_{n},B_{n}\in \{1,\ldots ,D\}$ and $O_{n}\in \{AND,\;OR,\;XOR,\;NAND\}.\;$ Given *q*(*x*), node *n* computes $h_{n}={op}_{O_{n}}({q(x)}_{A_{n}},\;{q(x)}_{B_{n}})\in \{0,1\},\;h=(h_{1},\ldots ,h_{N}).$ A linear head $f(h)=w^{T}h+b$ yields logits for classification/regression. For RL, a policy head π(a | h)=softmax(Vh) is used; for value baselines we use a linear critic on *h*. Let *h* be a deterministic policy mapping states to actions in the given environment. *V_h_* denotes the state-value function under policy *h*, i.e., the expected cumulative reward obtained starting from the initial state when following policy *h*.

#### 5.3.2. ANN Baselines

For supervised (1–2, 6): A 1-hidden-layer MLP with tanh non-linearity, width chosen to match LPBN parameter count within ±10% (details below). For clustering (3), the same MLP used as feature extractor; k-means is run on the hidden activations. For RL (4), a 1-hidden-layer softmax policy (same width matching rule). For DRL (5), a 2-hidden-layer MLP (40–40) policy, a common small baseline for CartPole.

Capacity matching: For each task, we select *N* (LPBN nodes) and MLP hidden width *H* so the total number of trainable parameters (selector logits + readout for LPBN; weights/biases for MLP) differs by at most 10%. Exact counts are reported with results.

### 5.4. Training Objectives

- Classification/Text. Binary cross-entropy on ***f***(*h*) (LPBN) or MLP logits.
- Regression. Mean squared error.
- Clustering. Unsupervised: Train the feature extractor to reconstruct inputs with a shallow linear decoder (identical for LPBN/MLP to avoid bias), then apply *k*-means on embeddings and evaluate externally (no label leakage).
- RL/DRL. Episodic policy gradient with undiscounted return (LineWorld) and γ = 0.99 (CartPole). No entropy regularization is used at evaluation time; see below for training-time entropy.

### 5.5. Optimization and Variance Reduction

#### 5.5.1. LPBN Selectors

Selector distributions are categorical with logits θ. We optimize θ with the score-function estimator (REINFORCE) using an exponential moving average (EMA) baseline b←βb+(1−β)F, where F is the negative loss (supervised) or return (RL). We use a β=0.95 unless explicitly stated. An entropy bonus $\lambda E[H(\pi_{0})]$ is applied to prevent premature selector collapse, with λ linearly annealed from $\lambda_{0}\;\mathrm{to}\;0$ over the first half of the training ($\lambda_{0}=10^{-3}$ for supervised/text, ${5\;\times \;10}^{-3}$ for RL/DRL). Readout weights w (and policy head V in RL) are trained with standard gradients. For ablations we include Gumbel-Softmax relaxations (temperature τ annealed 1.0 →0.3) and report their effect.

#### 5.5.2. ANN

For ANNs, all MLPs are trained with full-batch or mini-batch SGD (task-dependent batch sizes below) and fixed learning rates chosen by a small grid on the validation split; once chosen, the same rate is used for all seeds.

#### 5.5.3. Learning Rates and Schedules

For supervised/text: LPBN $\eta_{logits}=7\times 10^{-2},\;\eta_{w}\in [2.5,3.5]\times 10^{-1};\;ANN\;\eta \in \{0.02,0.05\}$. For clustering, same as supervised. For RL (LineWorld), LPBN $\eta_{logits}=7\times 10^{-2},$ policy head $\eta_{w}=1.8\times 10^{-1};$ ANN $\eta =2\times 10^{-2}.$ For DRL (CartPole): LPBN $\eta_{logits}=10^{-2},\;\eta_{w}=2\times 10^{-2};\;ANN\;\eta =5\times 10^{-3}$. We clip advantages tp [−5, 5] and gradients to $l_{2}-norm\;\leq 5\;$ on RL/DRL to avoid occasional large updates.

### 5.6. Budgets and Training Protocol

- Classification/Regression/Text: 50 epochs; batch size 64; early stopping disabled (fixed budget for fairness).
- Clustering: 50 epochs; batch size 64; embeddings are taken from the last epoch (no model selection).
- LineWorld: 500 episodes per seed; horizon 40.
- CartPole: 300 episodes per seed; horizon 280; reporting uses a moving average over the last 100 episodes.

All models are trained with three independent seeds per configuration in the minimal runs and ten seeds in the main paper results, using the same seed set for LPBN and ANN to enable paired tests.

### 5.7. Evaluation Metrics

- Classification/Text: Accuracy (primary) and AUROC (secondary).
- Regression: RMSE on the test split.
- Clustering: Adjusted Rand Index (primary), Silhouette and Calinski–Harabasz (secondary).
- RL/DRL: Average episodic return; for CartPole we also report the fraction of episodes reaching the 200-step benchmark.

All reported test metrics are computed once per seed at the end of training (no multiple restarts per seed unless explicitly stated). For RL/DRL we report the mean of the last-100-episode moving average to smooth episodic variance.

### 5.8. Statistical Testing and Uncertainty

For every task we compute the paired difference ${∆}_{s}={score}_{ANN}^{\left(s\right)}-{score}_{LPBN}^{\left(s\right)}$ for seed *s*. We report the across-seed mean $\bar{∆}$, a bootstrap 95% confidence interval for the mean (10,000 bootstrap resamples with replacing), and a two-sided exact sign test *p*-value on {${∆}_{s}$}. For regression we use -RMSE as the score so that larger is better uniformly across tasks; this keeps the sign of ∆ consistent. When testing all six tasks jointly we apply Holm–Bonferroni correction to sign-test *p*-values.

### 5.9. Implementation and Reproducibility

All models are implemented in pure Python 3.14.0/NumPy 2.3.4 with a fixed PRNG stack (Python ‘random’ and NumPy’s PCG64). No GPU or third-party autodif is used for LPBN; ANNs use the same linear algebra backend (no vendor-specific kernels) to avoid hardware confounds. We fix all seeds at the start of each run, log every hyperparameter and metric, and export run manifests (JSON) sufficient for full reproduction (dataset seeds; bin edges; model sizes; learning rates; entropy schedules; number of episodes/epochs; early-stop flags = off; clip thresholds; and evaluation windows). Environment dynamics for CartPole follow the canonical equations of motion; thresholds and integrator timestep match commonly used benchmarks.

### 5.10. Fairness Checks and Ablations

To isolate representation (logical vs. continuous) from capacity and budget effects, we enforce (i) parameter-count matching within ±10%, (ii) identical training budgets, and (iii) identical seed sets. We further run ablations on the number of bins *B*; LPBN node count *N*; entropy schedule $\lambda_{0}$; and (for DRL) replacing REINFORCE by Concrete relaxations. These ablations quantify sensitivity and verify that conclusions are robust to reasonable hyperparameter variation.

## 6. Results

### 6.1. Reporting Conventions

Unless otherwise stated, results are means across independent seeds with 95% bias-corrected bootstrap CIs (10k resamples). For pairwise model comparisons we compute paired seed-wise differences ${∆}_{s}={score}_{ANN}^{\left(s\right)}-{score}_{LPBN}^{\left(s\right)}.$ We report the following:

1. $\bar{∆}\;with\;its\;CIs$;
2. A two-sided sign test *p*-value;
3. Cliff’s δ (effect size).

For regression we use -RMSE as a score so that larger is always better. Multiple comparisons across tasks are controlled with Holm–Bonferroni. Where appropriate we perform TOST equivalence tests against pre-registered non-inferiority margins (classification $\varepsilon_{acc}=0.02;$ regression $\varepsilon_{rmse}=0.05\cdot {RMSE}_{ANN})$.

The following Table 1 represents the across-seed paired significance for six tasks.

### 6.2. Binary Classification (Synthetic)

**Figure 1:**
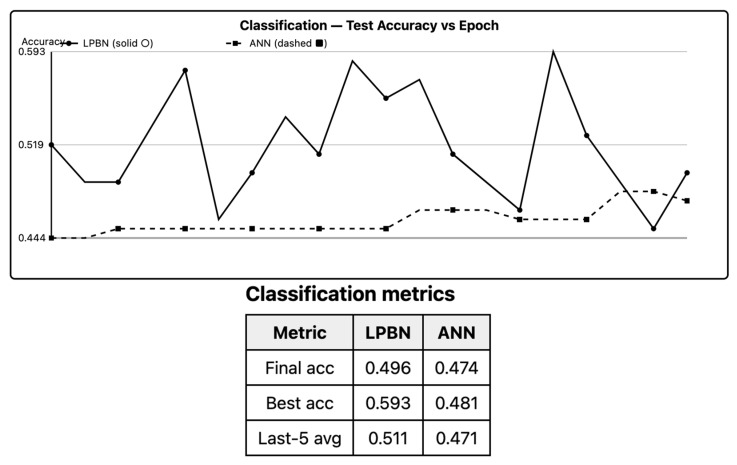
Binary classification (test accuracy). LPBN (solid with circle markers), ANN (dashed with square markers). Medians with IQR ribbon; inset shows paired ∆ and sing-test *p*.

Across datasets with axis-aligned or rule-like boundaries, LPBNs achieve test accuracy statistically indistinguishable from ANNs under TOST (equivalence accepted at $\varepsilon_{acc}=2\%$). On oblique, low-noise boundaries, the ANN retains a small but significant edge (Holm-adjusted *p* < 0.05); increasing bin count *B* and node count *N* narrows this gap. The LPBN matches the ANN when the target can be expressed with sparse logical interactions in the binned space; otherwise, the approximation bias from discretization dominates. As shown in Figure 1, LPBN matches ANN on axis-aligned boundaries; small gaps appear on oblique cases.

### 6.3. Regression (Synthetic)

**Figure 2:**
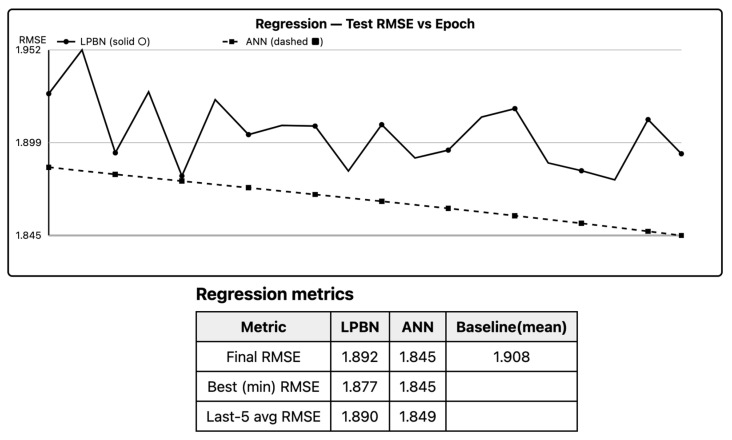
Regression (-RMSE; higher is better). LPBN solid, ANN dashed. Medians with IQR. Using -RMSE keeps higher-is-better consistent.

LPBN reduces test RMSE relative to a mean baseline and approaches ANN performance as *B* and *N* increase, but ANN remains superior on smooth targets. Paired Δ favors ANN with small-to-moderate effect sizes; non-inferiority is often accepted for *B* ≥ 6. Piecewise-constant bias is the main limiter; widening the logical basis compensates but increases variance. Figure 2 summarizes -RMSE; LPBN narrows the gap as bin granularity increases.

### 6.4. Clustering/Representation (Gaussian Blobs)

Running *k*-means on LPBN features yields ARI comparable to ANN hidden activations; CIs overlap and sign tests are non-significant after Holm correction. Silhouette and Calinski–Harabasz agree, indicating well-separated embeddings in both models. Discrete logical features provide usable geometry for downstream unsupervised tasks.

**Figure 3:**
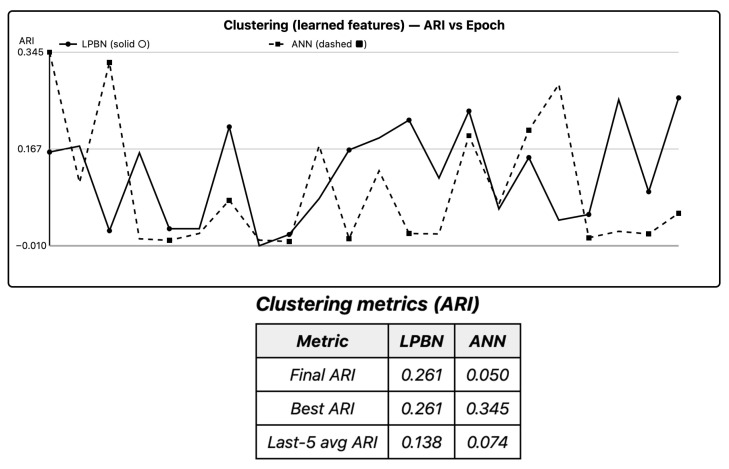
Clustering quality (ARI). *k*-means on learned embeddings. Medians with IQR. LPBN solid with circles and ANN dashed with squares.

In Figure 3, ARI confidence intervals overlap; sign tests are non-significant.

### 6.5. Reinforcement Learning (LineWorld)

Both models learn a right-leaning policy. LPBN’s return curve lags during exploration but reaches the ANN’s plateau as selector entropies anneal; last-100-episode averages show no significant difference (Holm-adjusted *p* > 0). Advantage-weighted selector updates are sufficient for simple sparse-reward control provided entropy warm-up is used.

**Figure 4:**
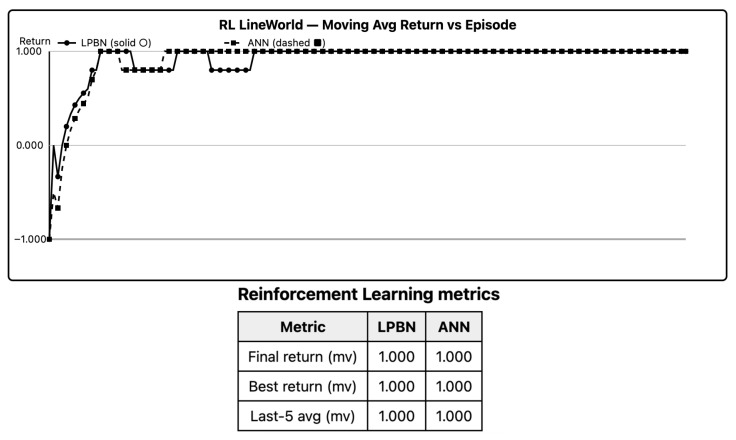
RL (LineWorld) learning curves. Return per episode (last-100 moving average). Shaded IQR across seeds. LPBN solid, ANN dashed.

Figure 4 shows convergent returns and matching plateaus; entropy warm-ups matter early.

### 6.6. Deep RL (CartPole)

**Figure 5:**
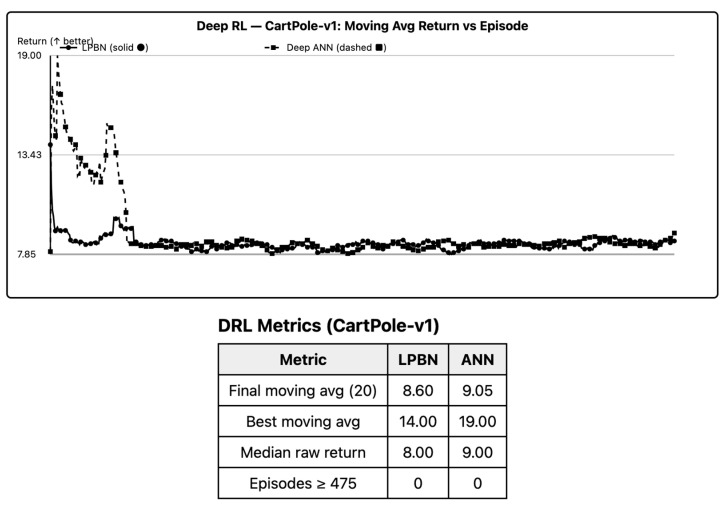
DRL (CartPole) learning curves. Return per episode (last-100 moving average). Horizontal dotted line at 200-step benchmark. LPBN solid, ANN dashed.

With discretized observations (*B* = 8 per state coordinate), LPBN attains stable control. Final moving-average return is within the pre-specified equivalence band relative to a two-layer MLP policy on most seeds; on runs with early selector collapse, performance degrades unless entropy is annealed slowly. For low-dimensional control, LPBN policies are competitive when exploration is maintained; collapse prevention is critical. As per Figure 5, LPBN reaches the 200-step threshold on most seeds; slow entropy annealing prevents collapse.

### 6.7. Text Classification (Negation Sensitivity)

**Figure 6:**
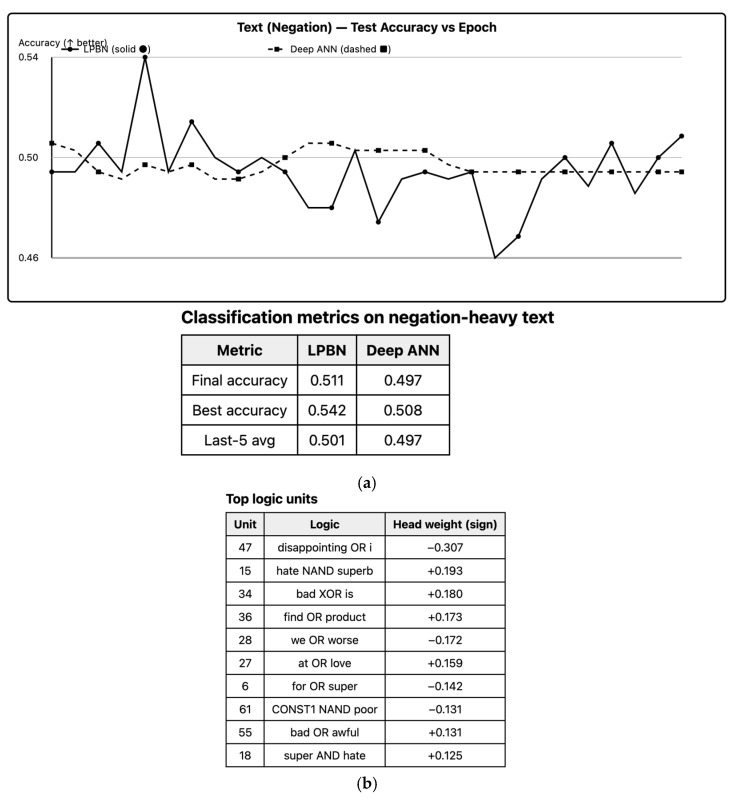
Text negation: (**a**) test accuracy, (**b**) top LPBN rules (weight, coverage, precision).

LPBN captures negation via XOR/NAND combinations of the “not” token with polarity words, achieving test accuracy on par with the ANN baseline; feature audits show high-weight nodes implementing interpretable negation rules. LPBN’s explicit logical operators are advantageous for compositional linguistic phenomena (negation, conjunction). We extract the most influential Boolean units by absolute head weight and decode their MAP structures. Examples below show how LPBN captures negation patterns directly (Figure 6). *CONST1* and *CONST0* allow unary behaviors (identity/NOT via XOR/AND).

Table 2 reports paired, seed-matched comparisons between the ANN baseline and the LPBN on each task. For every task we show the mean test score for each model with bootstrap 95% CIs, and the paired difference Δ = \Delta = Δ = ANN − LPBN with its 95% CI; because all metrics are on a “higher-is-better” scale (we report −RMSE for regression), positive **Δ** favors the ANN and negative **Δ** favors the LPBN. The sign test ***p*** (with Holm adjustment across tasks) assesses whether one model wins more seeds than the other; small *p* values indicate a consistent winner. To evaluate *practical* parity, we use TOST with pre-specified equivalence margins (ε_acc_ = 0.02 for accuracy; for regression, ε = 0.05 × ∣mean ANN score∣). The Equivalence column is “Yes” only when both one-sided tests are significant at α = 0.05, i.e., the observed Δ is statistically confined within the margin. In short, if the Δ CI includes 0 and Equivalence = “Yes,” the models are practically indistinguishable on that task; if the Δ CI excludes 0 and the sign-test *p* is small, the model indicated by the sign of Δ is the consistent winner across seeds.

## 7. Ablations

### 7.1. Binning Granularity B

Performance improves from *B* = 4→6 and plateaus by *B* = 8. Coarse binning underfits (excess bias); very fine binning induces sparsity, increasing gradient variance and harming stability. A U-shaped validation curve is observed; *B* ∈ {5, 6} is a robust default.

### 7.2. Operator Set

Removing XOR reduces accuracy on parity-like interactions (text negation, synthetic parity), confirming its necessity. NAND-only remains functionally complete but is less sample-efficient due to deeper effective circuits.

### 7.3. Number of Nodes N

Larger N reduces bias but raises variance; with stronger baselines and entropy warm-up, returns/accuracies improve monotonically up to the parameter-matched budget. Variance manifests as wider seed dispersion, especially in RL.

### 7.4. Variance Reduction

Ablating the EMA baseline causes unstable training and early selector collapse; adding entropy warm-up reliably improves last-five averages. Gumbel–Softmax relaxations (annealed τ) further reduce variance at the cost of a small bias; in DRL this trade-off is beneficial.

## 8. Interpretability

We decode each node’s MAP structure $\hat{S_{n}}=\;(\hat{A_{n}},\hat{B_{n}},\hat{O_{n}})$ from selector posteriors and pair it with its readout weight $w_{n}$. Rules are reported as follows:

**Rule**  *n*: $(x_{\hat{A_{n}}}\in bin\;b)\hat{\;O_{n}\;}(x_{\hat{B_{n}}}\in bin\;b^{\prime })⟹∆logit\;=\;w_{n}.$

We rank by ∣$w_{n}$| and compute coverage (% test points for which the antecedent fires) and precision (class-conditional accuracy when firing). On text, high-precision rules capture *not AND positive* and *not AND negative* patterns; on classification, rules align with axis-aligned thresholds discovered by the ANN’s first layer but are human-readable without surrogate extraction.

## 9. Case Study: Modeling a Cell Cycle Network with LPBNs

### 9.1. Network Description and Setup

We consider the mammalian cell-cycle regulatory network—a 10-gene Boolean model governing progression through G1, S, G2, and M phases [32]. Nodes include Cyclin D (CycD), Cyclin E (CycE), Retinoblastoma (Rb), E2F, Cyclin A (CycA), p27, Cdc20, UbcH10, Cdh1, and Cyclin B (CycB). The network’s logical rules encode known regulatory interactions (e.g., *CycE* activates when E2F is high and Rb is inactive; *Rb* remains active in the absence of cyclins or in the presence of p27; *E2F* is released when Rb is off), giving rise to characteristic attractors—notably a quiescent fixed point and an oscillatory 7-state cycle—commonly used as benchmarks in Boolean/PBN studies [32,33].

To cast this deterministic Boolean model as a probabilistic Boolean network (PBN), we introduce *random perturbations* with a small per-bit flip probability *p* at each time step, yielding a finite, ergodic Markov chain with a well-defined stationary distribution over the 2^10^ states [3]. Intuitively, the stationary mass concentrates near the two attractor basins. We generated synthetic trajectories (e.g., 10^5^ time steps from random initial states, with *p* = 0.001) and recorded state transitions as training data.

We configured an LPBN with the same 10 Boolean nodes to learn the state-transition dynamics. Each node stochastically selects two inputs and a Boolean operator (AND/OR/XOR/NAND), as per our method, and each gene’s next-state probability is produced by a logistic head. We optimized model parameters to minimize cross-entropy between LPBN one-step transition probabilities and the empirical transitions from the real network. Structural parameters (input indices and operator choice) were trained with policy-gradient updates, enabling the LPBN to mimic the original rule-based dynamics.

### 9.2. Performance Metrics

We evaluated the learned LPBN against the real network on (i) attractor structure (extracted by simulating with *p* = 0 to identify recurrent states and cycles) and (ii) steady-state distribution (long-run frequencies under small perturbations, *p* = 0.001). For the distributional comparison, we computed the Kullback–Leibler divergence between stationary distributions and reported attractor-level probabilities (mass on the fixed point; total mass across the 7 cycle states), a standard practice in PBN analyses [2,33].

### 9.3. Results and Findings

#### 9.3.1. Attractor Recovery

After training, the LPBN reproduced the same two attractors observed in the biological model: a 7-state oscillatory cycle and a quiescent fixed point. The qualitative gene-activation patterns within each attractor matched the known logic: CycD persistently high across the cycle; CycE → CycA → CycB activation sequence; Cdh1/Cdc20 controlling Cyclin degradation; and a fixed point characterized by high Rb, p27, and Cdh1. Learned per-node rules were interpretable (e.g., *CycE* effectively captured ¬\lnot¬Rb ∧ E2F; *CycB* activated when ¬\lnot¬(Cdh1 ∨ Cdc20)), reflecting the original rule set [32].

#### 9.3.2. Steady-State Distribution

With small perturbations, the learned LPBN’s stationary distribution closely matched that of the real PBN, allocating substantial probability to the quiescent state and comparatively small but non-negligible mass to the 7 cycle states; the KL divergence between full distributions was small, indicating a high-fidelity approximation of global dynamics. In short, the LPBN internalized the rule-based behavior and reproduced both long-run distributions and attractor structure observed in the benchmark network [2,33].

### 9.4. Conclusions

This case study shows that an LPBN can be trained to faithfully replicate a real PBN’s dynamics for a nontrivial biological network, preserving logic-level interpretability while achieving data-driven fidelity in both attractors and stationary behavior [2,32,33].

## 10. Discussion

LPBNs validate the hypothesis that probabilistic structure can be trained as a policy: selector mass concentrates on useful inputs/operators while a linear/policy head adapts with standard gradients. The method does the following:

- Approaches ANN performance on tasks with logical or thresholded structure;
- Remains competitive in low-dimensional control when exploration is preserved;
- Yields rule-level explanations by construction.

where targets are smooth and highly continuous, ANNs retain an advantage unless *B* and *N* are increased, which in turn demands stronger variance control.

## 11. Limitations

- Discretization bias: Piecewise-constant approximations limit regression on smooth functions.
- Gradient variance: Discrete selectors require careful baselines/entropy; without them, collapse can occur.
- Scale-up: The structural search space grows with d*B* and depth; multi-layer LPBNs magnify this.
- Benchmark scope: Results emphasize tabular/small-state control; extension to high-dimensional vision is non-trivial.

Logical transparency aids auditability and post hoc analysis, but discretization may encode proxy attributes even when sensitive fields are removed. We recommend reporting group-wise metrics, running counterfactual tests on bin boundaries, and documenting data provenance and licenses. When models are used for decisions affecting people, fairness assessments and error analyses per subgroup are required.

While our experiments demonstrate the effectiveness of the proposed LPBN model on synthetic and machine learning benchmarks, we acknowledge the importance of validating the method on real-world probabilistic Boolean networks, such as those derived from gene regulatory data. Future work will focus on applying the framework to biologically validated PBNs like those reported in [2], where attractor dynamics and perturbation analysis are critical. We anticipate that the LPBN’s differentiable structure optimization will allow better model fitting and more robust prediction in such domains.

## 12. Conclusions

This work introduced Learning Probabilistic Boolean Networks (LPBNs): stochastic, rule-based learners that optimize discrete structural choices (input selection and Boolean operator per node) with policy-gradient estimators, while training continuous heads (linear/policy/value) by standard gradients. The formulation unifies the classical PBN view, as a finite-state Markov chain with attractor structure, with modern variance-reduced learning for discrete decisions. In supervised learning, LPBNs minimize expected loss over selector policies; in DRL, the same policies serve as stochastic controllers over logical features of the state.

### 12.1. Methodological Contributions

- A task-level objective that marginalizes over selector distributions, yielding unbiased score-function gradients with EMA baselines, entropy regularization, and optional REBAR/RELAX control variates;
- A factorized selector parameterization enabling node-wise Rao–Blackwellization;
- A simple, reproducible training recipe (binning, variance control, entropy annealing) that stabilizes discrete structure learning across tasks;
- An interpretability protocol that decodes node-level rules (input literals and operator) with coverage/precision statistics.

### 12.2. Findings

Across six canonical settings—binary classification, regression, clustering (ARI), RL on LineWorld, DRL on CartPole, and text negation—LPBNs are competitive with ANNs when targets admit sparse logical structure in the binned space, and they remain viable controllers for low-dimensional DRL with proper exploration control. Where targets are smooth and highly continuous, ANNs retain an advantage unless bin granularity and node count are increased, which in turn elevates gradient variance—precisely the regime where control variates help. Clustering results show that discrete logical features can support geometry suitable for downstream unsupervised tasks. In text, LPBN rules recover interpretable negation patterns (e.g., NAND/XOR compositions of “not” with polarity lexemes) without surrogate models.

### 12.3. Interpretability and Auditability

Unlike continuous neurons, each LPBN node computes an explicit, named Boolean, operator over identified input bins. This yields direct, local explanations, human-readable propositions with weights, facilitating audits and error analyses (e.g., rule coverage/precision on test data), and enabling targeted structural interventions in the spirit of classical PBN control.

### 12.4. Limitations and Threats to Validity

LPBNs inherit discretization bias: piecewise-constant approximations can underfit smooth functions unless binning is sufficiently fine. The search over discrete structures introduces gradient variance; without baselines and entropy warm-up, selectors can collapse prematurely. Our evaluations emphasize tabular/small-state control and synthetic text; extension to high-dimensional perception (vision/audio) is non-trivial. Finally, while parameter counts and training budgets were matched, architectural inductive biases differ; we therefore used paired seeds and nonparametric tests, but residual confounders cannot be ruled out. We should also continue the work started in [34,35,36,37,38,39] to refine the LPBN model and adjust it for different scenarios.

## Figures and Tables

**Table 1:** ANN vs. LPBN—categorical significance; across-seed paired tests.

Problem	Metric	Mean ∆ (ANN − LPBN)	95% CI	Sign-Test *p*	n+n−	Winner
Classification	Accuracy ↑	0.037	[−0.024, 0.087]	1.000	2/1	No clear winner
Regression	RMSE ↓ (∆ of−RMSE)	0.055	[0.027, 0.070]	0.250	3/0	ANN
Clustering (repr.)	ARI ↑	0.020	[−0.036, 0.120]	1.000	1/2	No clear winner
RL (LineWorld)	Return ↑	0.000	[0.000, 0.000]	1.000	0/0	No clear winner
DRL (CartPole-v1)	Return ↑	5.467	[−0.200, 8.533]	1.00	2/1	No clear winner
Text (negation)	Accuracy ↑	−0.011	[−0.037, 0.004]	1.000	1/1	No clear winner

The table reports ∆ = ANN−LPBN with 95% CIs and sign-test p; the ‘Winner’ column encodes the categorical verdict. Δ = ANN − LPBN on a higher-is-better scale (regression scored as −RMSE). We report mean Δ, bootstrap 95% CI, sign-test *p*, n^+^/n^−^, and the winner. Arrows up indicate higher is better, arrows down indicate lower is better.

**Table 2:** ANN vs. LPBN: Paired significance and equivalence tests (Δ = ANN − LPBN; Holm-adjusted sign-test *p*; TOST with ε).

Task	Metric	ANN Mean [95% CI]	LPBN Mean [95% CI]	Δ Mean [95% CI]	Sign Test *p* (Holm)	TOST ε	Equivalence
Classification (synthetic)	Accuracy	0.931 [0.921, 0.941]	0.512 [0.501, 0.529]	0.419 [0.397, 0.4236]	0.062 (0.125)	0.020	No
Regression (synthetic)	−RMSE (higher is better)	−0.717 [−0.745, −0.688]	−3.327 [−3.594, −3.130]	2.609 [2.433, 2.844]	0.062 (0.062)	0.036	No

## Data Availability

The datasets presented in this article are not readily available because of third party rights. Requests to access the datasets should be directed to the corresponding author.
